# Finding our way home

**Published:** 2017-12-15

**Authors:** J Damon Dagnone

**Affiliations:** 1 Department of Emergency Medicine, Queen’s University, Ontario, Canada

**Figure:**
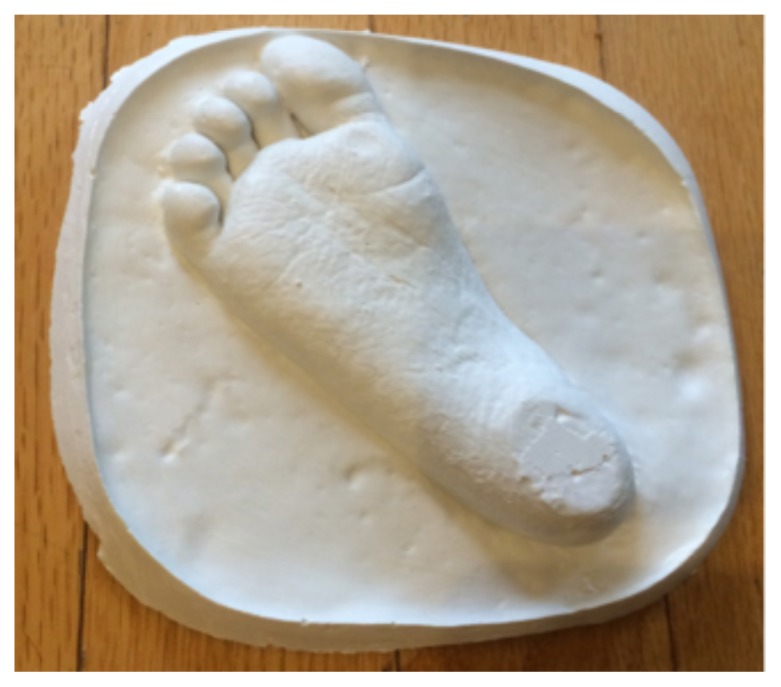


Eleven years ago, my wife and I lost our son Callum to cancer. He had just celebrated his 3^rd^ birthday at Sick Kids Hospital in Toronto where he was undergoing his 3^rd^ “rescue” stem cell transplant for his 6^th^ and final chemotherapy cycle, following the surgical removal of a brain tumor. The image above is a plaster imprint of Callum’s untouched foot, and was created on November 11^th^, 2006 – just moments after he died in the Paediatric Intensive Care Unit. Its tiny proportions, detailed imprinting, and delicate craftsmanship all powerfully convey its simple message. Protected permanently in our home, it is being shared in CMEJ to help tell my personal journey as a bereaved parent, loving husband, individual human being, and Emergency Room physician. It accompanies and amplifies Lester Liao’s commentary (in this issue) on “The physician as person framework.”

For my wife and me, the image of our son’s footprint represents many things. At the time of its creation, it represented a final keepsake created during a time of intense suffering. The imprint of his foot also captured one of the last remaining parts of our son that had not been affected by the ravages of cancer and struggles of his treatment – the invasive monitors, intravenous lines, tubes, and the destructive transformation of his physical appearance during the end stages of his illness. it represents for us our journeys together and alone.

Our first journey together was with Callum, starting at the time of his diagnosis, continuing through all of his treatments, and ending with his death. My wife and I asked our little boy to trust us that that he “had to get sick from the medicine to then get better.” We asked him to embrace the nurses, doctors, and everyone caring for him as we entered scary and dangerous places. We asked him to be his best self despite the debilitating chemotherapy that forced us into months of isolation confined mostly to a single barren bed in a small room within a hospital building far from home. To ask this of our baby knowing that we might lose the battle was torture for us – and yet our son showed us the way. He took us by the hands and led us on our journey in that hospital - with his smiles, his patience, and his quiet acceptance of his condition. During his illness, he made us the proudest parents on earth.

When my wife and I lost Callum, our world shattered and our new journey began without him. This second journey, also captured by his footprint, involves rebuilding our lives and it has been so very hard to walk this path we must. These last 11 years have been so difficult and unfair, but like the first journey we asked our Callum to take with us, we have no choice in this one either. Only this time, this new journey is so much lonelier with so much more heartache.

Prior to my son’s diagnosis, my wife and I shared a storybook life. I was married to my childhood sweetheart, I was a dad to two wonderful little boys, and I was entering the final year of my FRCPC Emergency Medicine residency. I couldn’t have dreamed of being more content with life than I was at the time – and I thought daily of how blessed I was. Cancer changed everything. As a dad, husband, physician, and person, I can attest that I have been forever changed. This experience has profoundly affected how I connect with people in the world and what kind of doctor I now strive to be. Callum’s journey reminds me that I need to continue walking my journey as he walked his.

